# Executive Dysfunction Assessed with a Task-Switching Task following Concussion

**DOI:** 10.1371/journal.pone.0091379

**Published:** 2014-03-11

**Authors:** Ulrich Mayr, Charlene LaRoux, Tyler Rolheiser, Louis Osternig, Li-Shan Chou, Paul van Donkelaar

**Affiliations:** 1 Department of Psychology, University of Oregon, Eugene, Oregon, United States of America; 2 Department of Human Physiology, University of Oregon, Eugene, Oregon, United States of America; 3 School of Health and Exercise Sciences, University of British Columbia – Okanagan, Kelowna, British Columbia, Canada; University College London, United Kingdom

## Abstract

Concussion frequently results in executive function deficits that can be specifically probed using task-switching tasks. The current study examined in detail the influence of concussion on task switching performance using both spatial and numerical stimuli. Individuals with concussion (n = 16) were tested within 48 hours of injury and 7, 14, and 28 days later. Healthy sex-, age-, height-, weight- and activity-matched controls (n = 16) were also tested at the same intervals. Switch costs were significantly greater in the participants with concussion than in the controls for both types of stimuli. By contrast, the global costs on non-switching trials were unaffected by concussion. We conclude that concussion has pronounced negative effects on the ability to switch task sets that generalize across task combinations (spatial or numerical) and that persist across at least a month after injury.

## Introduction

It is estimated that 1.6 to 3.8 million individuals suffer a sports-related concussion annually in the United States [1], [2]. Concussion are associated with a variety of symptoms including headache, balance problems, and neurocognitive deficits [3]. We have demonstrated previously that individuals with concussion frequently suffer deficits in the ability to maintain and allocate attention within and between tasks [4]–[6], suggesting that concussed athletes suffer from disruptions to executive function.

One means by which executive function can be effectively probed is by the use of task-switching paradigms. In such paradigms, subjects are required to actively switch the manner by which stimuli and responses are mapped to each other [7], [8]. Previous studies have demonstrated task-switching deficits in moderate to severe traumatic brain injury (TBI) [9]; and we have recently shown that concussed adolescents display task-switching deficits for up to two months post-injury [10].

Mayr & Bell [8] demonstrated in healthy young adults that switch costs were substantially smaller in magnitude for a non-spatial switch task using numbers compared to a more traditional spatial switch task using visual targets the location of which varies across trials. This observation suggests that processing during the spatial version of the task involves greater influence from ‘bottom-up’ factors inherent in the stimuli and thereby requires more careful, and time-consuming, control to maximize successful performance. Whether similar processes contribute to the task-switching deficits in concussion is currently not known.

Thus, in the present study we characterized task-switching deficits following concussion using both spatial and numerical stimuli across four time points over a one-month post-injury period. By this means, we were able to assess the generalizability and time course of functional recovery of task-switching deficits following concussion.

## Methods

### Participants

Sixteen individuals with concussion were identified from within the University of Oregon population and were tested within 48 hours of injury and 7, 14, and 28 days later. Sixteen healthy sex, age, height, weight and activity level matched controls were also tested at the same intervals. The two groups did not differ in on any of these variables (Table 1). All concussion participants were recruited for participation in the study after being identified as having a concussion by certified athletic trainers and/or attending medical doctors in the university intercollegiate athletic program or student health center. The source of injury included impacts to the head during a sporting activity, or accidental falls or collisions. For the concussed individuals who were athletes, controls were recruited from the same team/sport. The definition of concussion was based on the 3rd International Statement on Concussion in Sport as an injury caused by a direct blow to the head, face, neck or elsewhere in the body with an impulsive force transmitted to the head resulting in impaired neurologic function and acute clinical symptoms [11]. Exclusion criteria for both subject groups included: 1) a history of cognitive deficiencies, 2) a history of three or more previous concussions, 3) a loss of consciousness from the concussion lasting more than one minute, or 4) a previously documented concussion within the past 12 months. All participants provided written informed consent prior to partaking in the study and the Institutional Review Board at the University of Oregon approved the experimental protocol.

**Table 1:** Participant Characteristics.

Measure	Group	Mean (S.D.)	*T* test	*P* value
**Age, yr**	Control	21.3 (2.6)	0.64	0.49
	Concussion	21.9 (2.4)		
**Education, yr**	Control	16.3 (1.2)	0.15	0.83
	Concussion	16.6 (1.3)		
**Sex**	Control	13 M 3 F		
	Concussion	13 M 3 F		
**Height, cm**	Control	173.4 (15.7)	0.36	0.63
	Concussion	171.7 (17.2)		
**Weight, kg**	Control	79.8 (9.3)	0.53	0.41
	Concussion	82.3 (10.7)		

### Task

**Figure 1:**
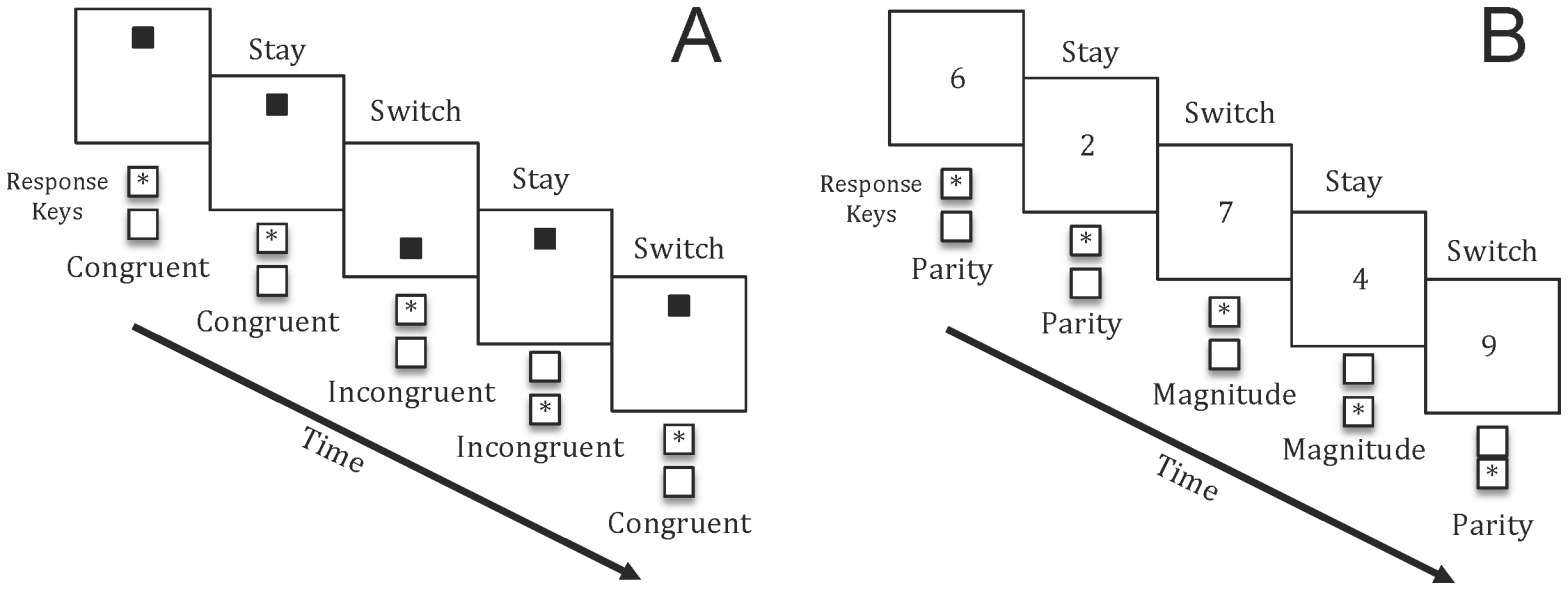
Task Paradigm. Trial events occurring during the spatial (A) and numerical (B) versions of the task-switching task. Participants responded to visual stimuli presented on a computer screen by pressing the button (*asterisk*) appropriate for the specific task contingencies.

The behavioral paradigm consisted of a spatial or numerical switching task (Figure 1). In the spatial version of the task, the participant sat in front of a computer monitor on which was presented a vertically oriented rectangle 10.3 cm×3.3 cm, within which a black 1 cm square appeared either at the top or bottom. In the “congruent” condition the participant was instructed to quickly and accurately indicate whether the square was at the top or bottom of the rectangle by pressing the “7” or “4” keys on a standard computer keyboard, respectively; whereas In the “incongruent” condition the participant was required to quickly and accurately press the “+” if the square appeared at the top or the “-“ button if the dot appeared on the bottom. After a correct response was made the subsequent square appeared 100 ms afterwards. By contrast, in the numerical switching task, a single digit between 1 and 9, excluding the number 5 was presented within a white square frame measuring 3.2 cm×3.2 cm at the center of the computer monitor. In the “magnitude” condition the participant was instructed to quickly and accurately indicate whether the number was greater than or less than 5 by pressing the “7” or “4” keys, respectively; whereas in the “parity” condition the participant was instructed to quickly and accurately indicate whether the number was odd or even by pressing the “+” or “−” keys, respectively. After a correct response was made and then was replaced 100 ms after the response by the subsequent number. In both paradigms, inaccurate responses resulted in the stimulus remaining on the screen. This feedback served to remind the participant of where they were within the sequence. Thus, for each paradigm the participant learned 2 sets of rules to respond to the same stimuli, and the responses for each set of rules was mapped onto 2 unique keys.

For both the spatial and numerical conditions, the participants completed the “simple” block of trials in which only one task constraint (i.e., congruent/incongruent or magnitude/parity) was followed as well as a “switching” block in which the task constraints were alternated across trials. In the spatial switch block, participants were required to switch from congruent to incongruent responses every 2^nd^ trial within the sequence, whereas in the numerical switch block, participants switched from magnitude to parity responses every 2^nd^ trial. During each session, separate blocks of trials with each combination of switch task (numerical vs. spatial) were completed in a counterbalanced order across subjects. Participants started each session with 10–20 practice trials in each condition followed by 80 experimental trials in each of the simple conditions and 160 experimental trials in each of the switching conditions. The total testing time was approximately 45 minutes.

### Data Analysis

The primary dependent variables of interest were reaction time and error rate. Reaction times were calculated as the time from onset of the stimulus to the time when the subject pressed one of the potential response keys. We omitted from the reaction time analysis trials in which errors were made and trials with reaction times greater than 5000 ms. The former accounted for ∼10% of the trials (see Results) and the latter <1%. The reaction times from the simple and switch blocks of trials were used to calculate the proportional ‘global’ and ‘switch’ costs for both the spatial and numerical conditions. Proportional global cost was calculated as the proportional difference in reaction times between simple blocks of trails and the ‘stay’ trials (i.e. trials in which the subject maintained their current mode of responding) during the switch blocks of trials. By contrast, the proportional switch cost was calculated as the proportional difference in reaction times between simple blocks of trails and ‘switch’ trials (i.e. trials in which the subject switched from responding in one mode to the other) during the switch block of trials. Error rate was calculated as the percentage of trials within a condition in which the participant responded incorrectly by pressing the wrong response key. Data were analyzed using 2 (subject group: concussed/controls) ×2 (condition: spatial/numerical) ×4 (time: 48 hours, 7, 14, and 28 days) mixed model analyses of variance for each dependent variable (switch cost, global cost, error rate). For all omnibus tests, significance was set at p<.05 with follow up Bonferroni-corrected pairwise comparisons. All statistical analyses were performed using SPSS version 20 (SPSS Inc, Chicago, Illinois).

## Results

### Reaction Time

The mean reaction times across the simple and switch versions of both the spatial and numerical versions of the task are displayed in Table 2. These values were used to calculate both the proportional switch and global costs that were analyzed statistically.

**Table 2:** Reaction times (ms).

Group	Condition	Task	Mean Reaction Time (S.D.)			
			2 Days	7 Days	14 Days	28 Days
Control	Spatial	Congruent	339 (42)	332 (35)	337 (41)	327 (32)
		Incongruent	357 (48)	358 (45)	349 (48)	345 (39)
		Stay	445 (57)	407 (49)	375 (43)	372 (47)
		Switch	642 (84)	587 (74)	583 (69)	602 (79)
Concussion	Spatial	Congruent	415 (52)	394 (45)	368 (42)	362 (41)
		Incongruent	434 (56)	416 (47)	382 (47)	379 (45)
		Stay	544 (64)	477 (53)	410 (47)	419 (49)
		Switch	1087 (116)	931 (95)	825 (92)	778 (79)
Control	Numerical	Magnitude	544 (52)	548 (57)	549 (56)	543 (57)
		Parity	556 (56)	549 (61)	542 (52)	548 (51)
		Stay	604 (72)	589 (71)	579 (78)	572 (68)
		Switch	881 (86)	850 (79)	738 (69)	817 (72)
Concussion	Numerical	Magnitude	601 (74)	592 (69)	587 (65)	583 (63)
		Parity	610 (73)	584 (62)	593 (68)	589 (67)
		Stay	684 (71)	624 (70)	623 (67)	608 (65)
		Switch	1173 (128)	1025 (104)	1032 (111)	969 (91)

### Switch Cost

**Figure 2:**
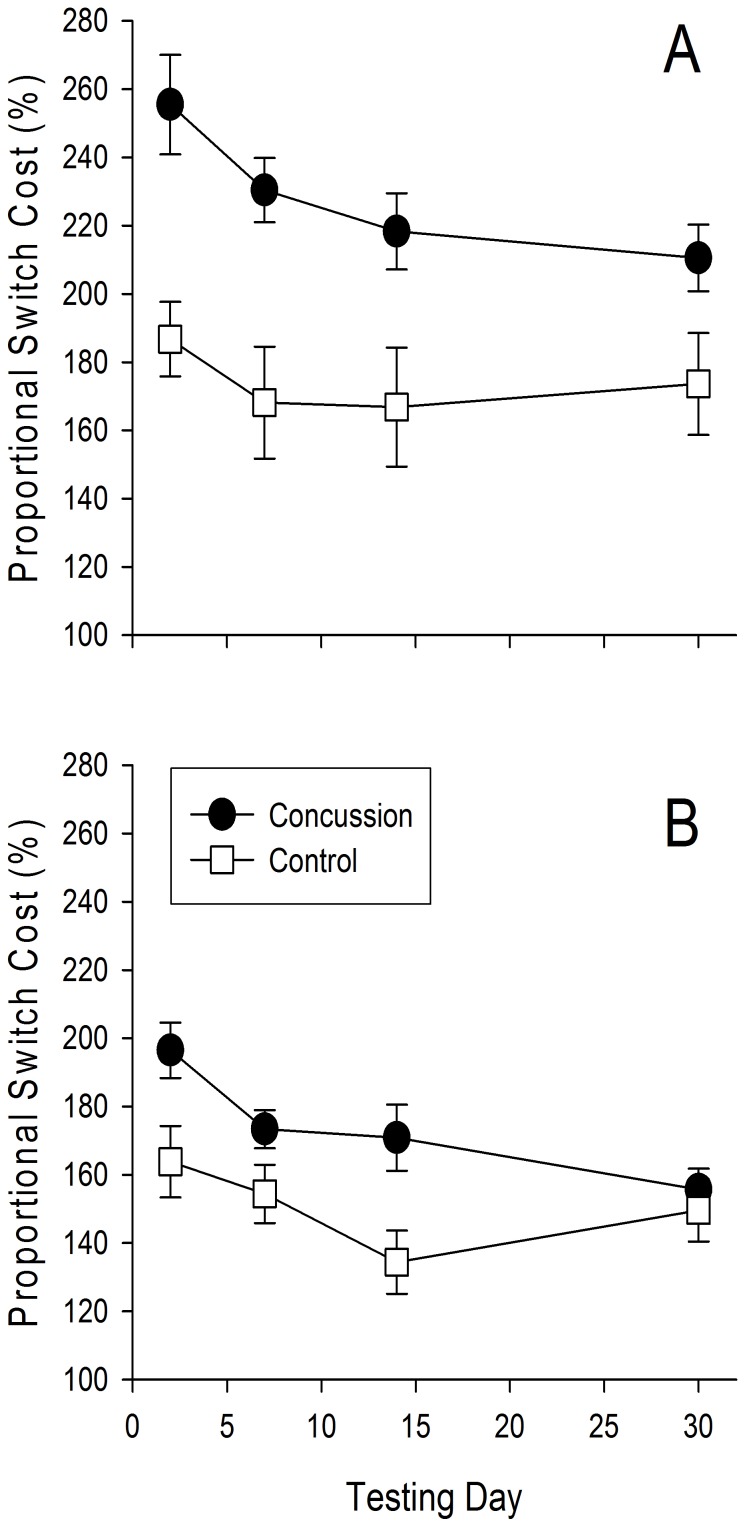
Switch Cost. Switch cost across the 4 testing days in the spatial (A) and numerical (B) versions of the switch task for the participants with concussion (*black circles*) and controls (*white squares*). Error bars, *1 intersubject SE*.

Figure 2 illustrates the proportional switch cost in the spatial and numerical conditions across all four testing days. Statistical analysis revealed that this variable was greater overall in the concussed participants (F[1,255] = 23.73, p = .0004) and in the spatial version of the task (F[1,255] = 18.91, p = .0007). There was also a significant day effect (F[3,255] = 4.44, p = .006), but no significant group × day (F(3,255) = 0.13, p = .73) or condition × day interactions (F(3,255) = 0.42, p = .34)). Taken together, this demonstrates that switch costs were increased overall by concussion and improved as a result of a combination of injury recovery and repeated exposure to the task. The lack of significant group by day interaction implies that, despite the improvement across days, the group differences observed in switch costs immediately after the injury remained 1 month later.

### Global Cost

**Figure 3:**
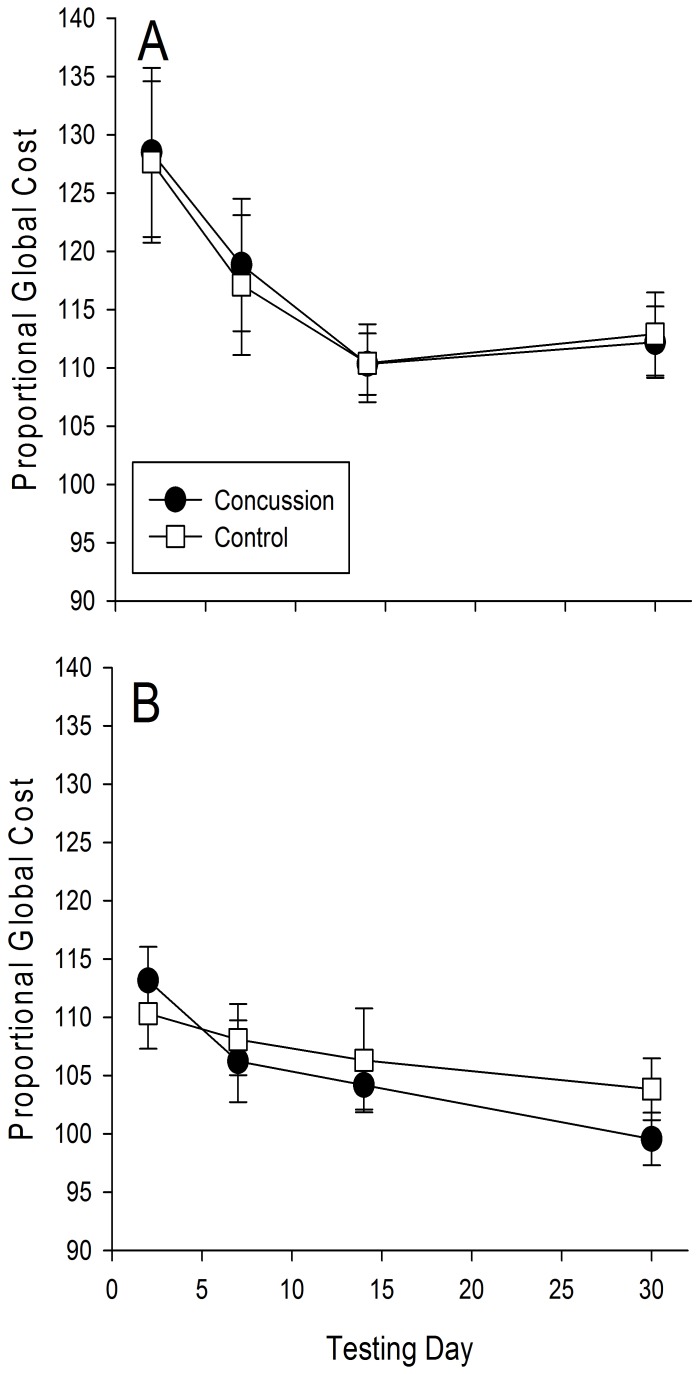
Global Cost. Global cost across the 4 testing days in the spatial (A) and numerical (B) versions of the switch task for the participants with concussion (*black circles*) and controls (*white squares*). Error bars, *1 intersubject SE*.

Figure 3 illustrates the proportional global cost in the spatial and numerical conditions across all four testing days. Statistical analysis revealed a significant day effect (F[3,255] = 4.58, p = .006), but no significant group (F[1,255] = 0.65, p = .38) or condition effects (F[1,255] = 0.25, p = .58). In addition, none of the interactions were significant (group × day (F[3,255] = 0.16, p = .72); condition × day (F[3,255] = 0.89, p = .25)). Thus, as with the switch costs, participants improved overall in their global costs with repeated exposure to the task. However, concussion did not adversely affect this component of executive function.

### Error Rate

Analysis of the error rate data demonstrated that there were no significant main effects or interactions. Error rates were approximately 10% for both controls and concussed participants in both the spatial and numerical tasks and this did not change significantly across the 4 testing days.

## Discussion

In the present experiment we examined the executive function deficits induced by concussion using a task-switching paradigm. Our results showed that participants with concussion had significantly larger switch costs than controls. By contrast, global costs were similar in both groups. These deficits in switching partially generalized across the spatial and numerical conditions indicating that the deficit observed in concussed participants is due in part to difficulty in the process of switching itself irrespective of the form of switch task. Indeed, the overall difference across conditions was consistent with previous research [8], indicating that, relative to controls, concussion does not differentially influence the ability to perform the task under the spatial and non-spatial contexts. In addition, the switch cost differences between participants with concussion and controls were maintained throughout the 1-month testing period. Taken together, the results indicate that task-switching effectively probes the executive dysfunction associated with concussion and suggests that the brain regions involved in executive function may be particularly susceptible to damage or disruption from this injury.

We have previously demonstrated that participants with concussion have small but pervasive deficits in the spatial distribution of attention as probed by the Attentional Network Test (ANT) [5], [6]. In particular, although concussion did not affect the alerting component of attention, both the orienting and executive components were substantially modulated by the injury. Furthermore, the results indicate that these dissociable attentional networks are subject to differing rates of recovery. Specifically, the deficits observed in the ability to orient attention resolved within the first week post-injury, whereas the executive component deficits were still present 30 days post-injury. In addition, we have observed similar subtle, but systematic, deficits in the ability to disengage attention [12], distribute attention across time [13], and countermand saccadic eye movements [4]. Although the deficits we have observed in these tasks are statistically significant, they are, nevertheless, quite subtle. This may be due to the automaticity inherent in the experimental tasks themselves [14]. In particular, because of their nature, such tasks are thought to automatically engage executive function processes. By contrast, task-switching requires active, top-down regulation to facilitate successful performance and therefore, is a more sensitive probe of executive dysfunction after concussion as can be observed by the larger differences in performance between patients and controls in the present study. Analogous task switching deficits have been observed previously in moderate to severe TBI [9]. Moreover, we have recently demonstrated that adolescents with concussion have task switching deficits in the spatial version of this task that persist up to 2 months after the injury [10].

Given the marked differences in performance between the participants with concussion and controls, we can ask what implications this has with respect to the network of brain areas that contribute to the processing underlying task switching. Recent brain imaging, lesion, and TMS studies have demonstrated that a number of medio-frontal sites play a key role in task switching performance. Rushworth and colleagues [15] showed that the medial frontal cortex (MFC) is transiently activated when a subject switches between the two stimulus-response mappings in a task-switching task. This activation denotes the processing associated specifically with the local costs of switching itself, not the global costs of being in the context of task-switching. This same group has demonstrated that lesions to cingulate cortex indirectly contribute to task switching deficits due to its normal function related to attention to action and voluntarily initiated action [16], [17]. In addition to medio-frontal areas, the lateral prefrontal cortex also appears to contribute to task switching performance. Furthermore, Mayr and colleagues [8] demonstrated that individuals with left prefrontal lesions suffered increased reaction times during switching, analogous to the increased switch costs observed in the participants with concussion in the current study.

In conclusion, we have demonstrated that individuals suffering from recent concussion display an increased cost of switching between tasks within the first 48 hours of injury relative to healthy matched controls, and this increased switch cost is maintained throughout a 1 month post-injury testing period. Conversely, there is no significant difference in the global cost to reaction times between concussion and control participants while performing trials in which no switching is required. Taken together, this suggests that tasks which engage active executive function processes may be especially sensitive to the effects of concussion. Given this, with appropriate baseline testing, tasks of this nature may be useful in clinical protocols to initially diagnose and manage the recovery of function following concussion more objectively.
